# Methodology for the adolopment of recommendations for the treatment of rheumatoid arthritis in the Kingdom of Saudi Arabia

**DOI:** 10.1186/s12874-023-02031-2

**Published:** 2023-10-10

**Authors:** Joanne Khabsa, Sally Yaacoub, Mohammed A. Omair, Hanan Al Rayes, Elie A. Akl, the KSA 2021 ACR RA adolopment working group, Sultana Abdulaziz, Ghada A. Al Janobi, Abdulaziz Al Khalaf, Bader Al Mehmadi, Mahasin Al Nassar, Faisal AlBalawi, Abdullah S. AlFurayj, Ahmed Hamdan Al-Jedai, Haya Mohammed Almalag, Hajer Yousef Almudaiheem, Ali AlRehaily, Mohammed A. Attar, Lina El Kibbi, Liana Fraenkel, Hussein Halabi, Manal Hasan, Jasvinder A. Singh

**Affiliations:** 1 AUB Grade Center, American University of Beirut Medical Center, Beirut, Lebanon; 2 Clinical Research Institute, American University of Beirut Medical Center, Beirut, Lebanon; 3 Rheumatology Unit, Department of Medicine, King Saud University, Riyadh, Saudi Arabia; 4 Division of Rheumatology, Department of Internal Medicine, Prince Sultan Military Medical City, Riyadh, Saudi Arabia; 5 Department of Internal Medicine, American University of Beirut, Riad-El-Solh, P.O. Box: 11-0236, Beirut, 1107 2020 Lebanon; 6 Department of Health Research Methods, Evidence, and Impact (HEI), McMaster University, Hamilton, ON Canada

**Keywords:** Adaptation, Adolopment, Contextualization, Rheumatoid arthritis, American College of Rheumatology, Kingdom of Saudi Arabia, Glucocorticoids, Disease modifying anti-rheumatic drugs

## Abstract

**Background:**

Currently, there are no guidelines for the treatment of rheumatoid arthritis (RA) tailored to the context of the Kingdom of Saudi Arabia (KSA). Adaptation of guidelines accounts for contextual factors and becomes more efficient than de novo guideline development when relevant, good quality, and up-to-date guidelines are available. The objective of this study is to describe the methodology used for the adolopment of the 2021 American College of Rheumatology (ACR) guidelines for the treatment of RA in the KSA.

**Methods:**

We followed the ‘Grading of Recommendations Assessment, Development and Evaluation’ (GRADE)-ADOLOPMENT methodology. The adolopment KSA panel included relevant stakeholders and leading contributors to the original guidelines. We developed a list of five adaptation-relevant prioritization criteria that the panelists applied to the original recommendations. We updated the original evidence profiles with newly published studies identified by the panelists. We constructed Evidence to Decision (EtD) tables including contextual information from the KSA setting. We used the PanelVoice function of GRADEPro Guideline Development Tool (GDT) to obtain the panel’s judgments on the EtD criteria ahead of the panel meeting. Following the meeting, we used the PANELVIEW instrument to obtain the panel’s evaluation of the process.

**Results:**

The KSA panel prioritized five recommendations, for which one evidence profile required updating. Out of five adoloped recommendations, two were modified in terms of direction, and one was modified in terms of certainty of the evidence. Criteria driving the modifications in direction were valuation of outcomes, balance of effects, cost, and acceptability. The mean score on the 7-point scale items of the PANELVIEW instrument had an average of 6.47 (SD = 0.18) across all items.

**Conclusion:**

The GRADE-ADOLOPMENT methodology proved to be efficient. The panel assessed the process and outcome positively. Engagement of stakeholders proved to be important for the success of this project.

**Supplementary Information:**

The online version contains supplementary material available at 10.1186/s12874-023-02031-2.

## Background

The development of new guidelines is time- and resource-intensive. Alternatively, guideline developers can adopt existing recommendations, or adapt them to their own context [1]. When relevant, good quality, and up-to-date guidelines are available, adoption and adaptation become more efficient than development of new guidelines [2]. Some recommendations are context sensitive, i.e., their strength and/or direction are likely to be affected by contextual factors such as resources needed and acceptability. For such recommendations, adaptation accounting for contextual factors would lead to better applicability and subsequent uptake compared to adoption.

The ‘Grading of Recommendations Assessment, Development and Evaluation’ (GRADE)-ADOLOPMENT approach is increasingly being used to build on existing guidelines [3]. Adolopment includes the identification and prioritization of existing guidelines, the evaluation and completion of GRADE Evidence to Decision (EtD) tables for each recommendation, and final adoption, adaptation, or de novo development of recommendations. This approach has been applied in the region for a number of guideline topics [2, 4–9].

Currently, there are no guidelines for the treatment of rheumatoid arthritis (RA) tailored to the context of the Kingdom of Saudi Arabia (KSA). Management follows recommendations of both the American College of Rheumatology (ACR) and the European League Against Rheumatism (EULAR) [10, 11]. Yet, there are relevant differences in the KSA healthcare system and population that may affect certain recommendations. First, the KSA’s healthcare system consists of several sectors that report to different authorities and have their own resources, such as infusion units and medication formulary. Additionally, access to healthcare varies among different segments of the population, such as national civilians, military staff, and expatriates. While national civilians access the public healthcare system for free, expatriates can access only the private system through their insurance plans. The military staff has access to military hospitals. In addition, the availability of different types of medications varies by hospital [11].

Recently, the ACR published the 2021 update of guidelines for the treatment of RA [10]. The objective of this paper is to describe the methodology used for the adolopment of the 2021 ACR guidelines for the treatment of RA in the KSA [12].

## Methods

**Fig. 1:**
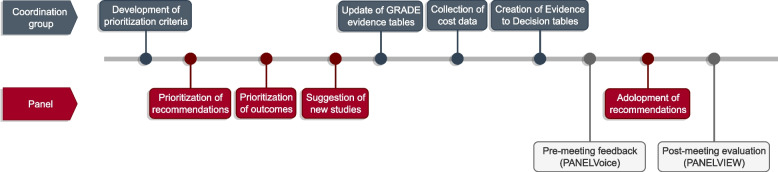
Overview of the methodology

We followed the GRADE-ADOLOPMENT methodology [3]. This methodology builds on the GRADE EtD framework, in that it uses the EtD criteria that determine the direction and strength of a recommendation to allow for the development of context-specific recommendations. Hence, the completion of GRADE EtDs for each guideline recommendation is a central element of this approach [3]. We provide an overview of the methodology in Fig. 1. In the subsequent sections, we describe the source guideline, groups involved, the process for prioritization of recommendations and outcomes, sources of data, the adolopment process and its evaluation.

### The source guideline

The 2021 American College of Rheumatology (ACR) guidelines for the treatment of RA includes 43 recommendations addressing questions on treatment with disease-modifying antirheumatic drugs (DMARDs) (24 recommendations), use of glucocorticoids (4 recommendations), and use of DMARDs in certain high-risk populations (15 recommendations). The development of the guidelines included the use of GRADE evidence tables and graded recommendations while accounting for the balance of benefits and harms, the certainty of the evidence, patient values and preferences, and resource use. While the methodology builds on the key principles of GRADE and includes many of the components of the GRADE EtD framework in a modified decision support voting mechanism, it does not explicitly include the production of an EtD table for each recommendation [13].

### Groups involved

The project involved a coordination group and a guideline panel. The coordination group included the two content experts leading the project (MAO, HAR) and three methodologists (EAA, JK, and SY). That group facilitated the handling of both logistical and methodological aspects of the work. The panel included representatives from different stakeholder groups, including rheumatologists, a pharmacist, a patient representative, and policymakers. The rheumatologists included two international experts (the chair of the source guideline and the chair of the 2015 ACR guideline [4]). The panel included 19 members from the different Saudi regions and type of practice (i.e., both governmental and private). Table 1 presents the characteristic of panel members. There were multiple touchpoints between the coordination team and the panel through email communication and online meetings.

**Table 1:** Characteristics of panel members (*N* = 19)

Characteristics of panel members	n (%)
Sex	
Female	9 (47)
Male	10 (53)
Stakeholder group	
Rheumatologist	15 (79)
Policymaker	2 (11)
Patient representative	1 (5)
Pharmacist	1 (5)
Years of experience in the above role (median, IQR)	16 (8 – 22)
Kingdom of Saudi Arabia region	
Central region (Riyadh and Al-Qassim Regions)	11 (58)
Western Region (Mecca Region)	4 (21)
Eastern Region	2 (11)
Not applicable (international experts)	2 (11)
Type of practice	
Governmental	15 (79)
Private	3 (16)
Not applicable (patient representative)	1 (5)

### Prioritization of recommendations and outcomes

Given the limited time and resources, the coordination group chose to prioritize five recommendations to be adapted from the source guideline. First, we developed a list of five adaptation-relevant prioritization criteria (Table 2). The selection of these criteria was based on (1) two reviews of the literature on prioritization for guideline development [14, 15], (2) a review of handbooks by 23 guideline-producing organizations on guideline adaptation (unpublished), and (3) expert input from the rheumatologist members of the coordination group. Second, through an online survey, we asked panelists to rate the priority of each of the source recommendations based on each criterion on a scale of 1 to 5 (1 indicating lowest priority, and 5 indicating highest priority). Third, we produced a list of ranked recommendations using the average priority score for each recommendation. The latter consisted of the average priority score across the five criteria for prioritization and across all respondents. Finally, we selected for the adaptation effort the five recommendations with the highest priority scores.

**Table 2:** Five selected criteria for prioritization of recommendations to be adapted

• Difference in resource use of the considered interventions in the KSA context relative to the original context
• Difference in feasibility of the considered interventions in the KSA context relative to the original context
• Difference in acceptability of the considered interventions in the KSA context relative to the original context
• Difference in impact on equity of the considered interventions in the KSA context relative to the original context
• Difference between current practice in the KSA context and the recommended intervention

As for the prioritization of outcomes, we reviewed the priority list set by the ACR and the systematic review on the topic [16], i.e., disease activity as a critical outcome; and physical function, radiographic progression, quality of life, and adverse events as important outcomes. The panel adopted these outcomes and their valuation after a discussion including the patient representative.

### Sources of data

**Fig. 2:**
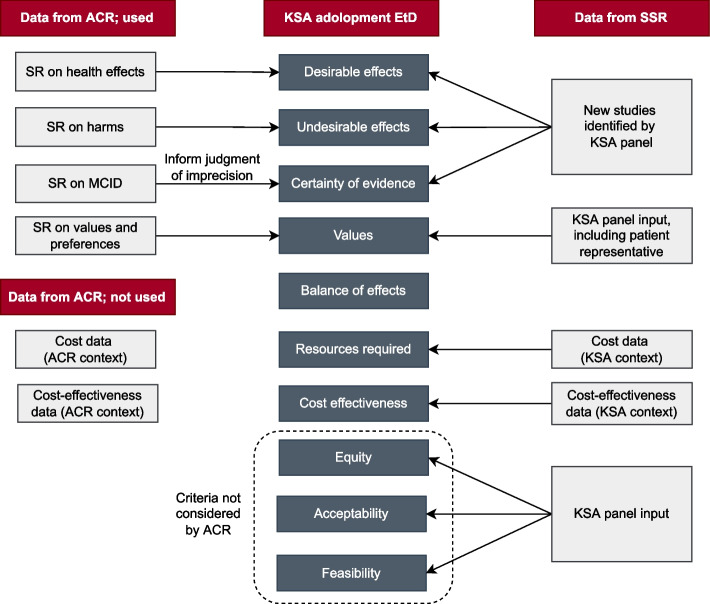
Sources of data for the Kingdom of Saudi Arabia adolopment effort. Abbreviations: ACR, American College of Rheumatology; EtD, Evidence to Decision; KSA, Kingdom of Saudi Arabia; MCID, minimal clinically important difference; SSR, Saudi Society of Rheumatology; SR, systematic review

Figure 2 presents the sources of data for the adolopment effort. Specifically:

- Evidence on health effects: we judged that a formal update of the evidence reports published by the ACR guidelines was not needed given the short timeframe between the publication of the source guideline and the adaptation project. Instead, we asked panelists to suggest any new studies published since the source guideline. If that was the case, we followed standard systematic review methodology to assess eligibility, abstract data and update the analyses, as described by the Cochrane handbook (e.g., duplicate and independent screening and data abstraction) [17]. Then, the three methodologists updated the GRADE evidence tables from the source guideline.
- Values and preferences: as mentioned above, the panel adopted the source guideline's ratings after consideration of the relevant systematic review on the topic [16] and the input of the patient representative.
- Cost data: we collected relevant cost data using two sources: the Saudi Food Drug Authority (SFDA) for the private sector [18] and the National Unified Procurement Company (NUPCO) for the governmental sector [19].
- Other contextual factors (i.e., impact on equity, acceptability, feasibility): as we did not conduct a formal assessment of these factors, we relied on the panel’s personal knowledge and experience.

### Adolopment process

The methodologists created EtD tables using the sources of data mentioned above. Before each recommendation meeting, we electronically shared the EtDs with the panelists and obtained their preliminary feedback and judgments using the PanelVoice function of GRADEPro Guideline Development Tool (GDT) (https://www.gradepro.org/). During the online panel meetings, the panelists decided on a judgment for each criterion of the EtD after reviewing the preliminary feedback and judgments, and discussion. When consensus could not be reached, we went with the majority vote and noted the vote results in the EtD.

### Evaluation

After completing the panel meetings, we invited the panelists to complete an online survey to evaluate the adaptation effort. We used the PANELVIEW instrument, which consists of 34 items relating to the guideline process, methods and outcomes [20]. The PANELVIEW instrument covers, among others, administration, training, conflict of interest, group composition, group interaction, considering the evidence and contributing through expertise, and formulating the recommendations. Panelists were asked to indicate their agreement on each item using a 7-point Likert scale. We generated a mean score and its standard deviation (SD) for each item. We also generated a mean score and its standard deviation across items.

## Results

We conducted the adolopment process over a period of four months. Additional file 1 shows the detailed timeline.

### Adoloped recommendations

Table 3 presents the five adoloped recommendations, along with EtD criteria influencing any modification from the source recommendations. Out of five adoloped recommendations, two remained unmodified (#2 and #4). To note that for recommendation #2, the KSA panel considered the increased risk of hemolysis with the use of hydroxychloroquine (HCQ) in the KSA due to the relatively high prevalence of glucose-6-phosphate dehydrogenase (G6PD) deficiency in this population [21]. However, it did not lead to a modification in recommendation #2, as the panel still had higher safety concerns for sulfasalazine (SSZ).

**Table 3:** Adoloped recommendations and Evidence to Decision (EtD) criteria driving any modification

Adoloped recommendations	Difference in relation to the source recommendations		EtD criteria driving any modification
	Direction	Certainty	
1. Initiation of treatment in DMARD-naive patients with low disease activity, sulfasalazine is conditionally recommended over methotrexate (conditional recommendation; based on very low certainty evidence)	Modified (from conditional in favor of SSZ)	Unmodified	• Balance of effects • Cost • Acceptability in relation to dosing convenience^a^
2. Initiation of treatment in DMARD-naive patients with low disease activity, hydroxychloroquine is conditionally recommended over other csDMARDs (conditional recommendation; based on very low certainty evidence)	Unmodified	Unmodified	
3. Initiation of a csDMARD without short-term (< 3 months) glucocorticoids is conditionally recommended over initiation of csDMARD with short-term glucocorticoids (conditional recommendation; based on very low certainty evidence)	Modified (from conditional against glucocorticoids)	Unmodified	• Valuation of outcomes • Balance of effects
4. For patients taking oral methotrexate who are not at target, a switch to subcutaneous methotrexate is conditionally recommended over addition/switch to alternative DMARD(s) (conditional recommendation; based on very low certainty evidence)	Unmodified	Unmodified	
5. For patients taking methotrexate plus a bDMARD or tsDMARD who wish to discontinue a DMARD, gradual discontinuation of methotrexate is conditionally recommended over gradual discontinuation of the bDMARD or tsDMARD (conditional recommendation; based on very low certainty evidence)	Unmodified	Modified (from very low)	

*Abbreviations*: *EtD* Evidence to Decision, *DMARD* disease-modifying antirheumatic drug, *csDMARD* conventional synthetic disease-modifying antirheumatic drug, *bDMARD* biological disease-modifying antirheumatic drug, *tsDMARD* targeted synthetic disease-modifying antirheumatic drug

^a^Low rate of adherence to medications in the Kingdom of Saudi Arabia. MTX administered weekly, SSZ administered twice daily

For recommendation #1, the panel modified the direction in relation to the balance of effects, cost, and acceptability in relation to dosing convenience. Specifically, the KSA panel judged that the undesirable effects of methotrexate (MTX) were less relevant to the KSA setting (particularly in terms of alcohol-induced hepatoxicity). This tilted the balance of desirable and undesirable effects in favor of MTX. Also, the panel took into consideration that the cost of a 12-week treatment course of SSZ is substantially higher than that of MTX. In addition, the panel judged that a generally low rate of adherence to medications in the KSA favored MTX (administered weekly) over SSZ (administered twice daily) (acceptability criterion).

For recommendation #3, the panel modified the direction in relation to the valuation of outcomes and the balance of effects. Specifically, the KSA panel highly valued rapid alleviation of symptoms at the time of diagnosis and during flares. Accordingly, the KSA panel judged that the balance of effects “probably favors” short-term (< 3 months) glucocorticoids when initiating a csDMARD. Still, the KSA panel acknowledged a few challenges with glucocorticoid treatment and detailed them in the rationale as a note of caution to users.

For recommendation #5, we updated the evidence to incorporate a newly published study. As a result, the certainty of evidence increased from very low to moderate, but did not lead to a modification in the strength or direction of the recommendation.

### Evaluation results

Out of 19 panelists, 13 filled the PANELVIEW survey. Additional file 2 presents the survey data, and Additional file 3 presents the mean and its standard deviation for each of the 34 evaluation questions. The mean scores on the 7-point scale ranged from 6.08 (for the items ‘adequate time was allotted for the final panel meeting for all guideline questions to be discussed and recommendations to be formulated’ and ‘the panel was given sufficient opportunity to be involved in the prioritization of questions and scoping of the guideline’) to 6.85 (for the item ‘I would participate in this guideline development process again’). There was a high ‘overall satisfaction’ with the guideline development process (mean score = 6.46). The mean score had an average of 6.47 (SD = 0.18) across all items.

## Discussion

In this paper, we described the methodology used for the adolopment of the 2021 ACR guidelines for the treatment of RA in the KSA. The process followed the GRADE-ADOLOPMENT methodology, and involved prioritization of recommendations and outcomes, and construction of EtDs for each prioritized recommendation. We populated the EtDs with data from the source guideline and with contextual data from the KSA.

We engaged relevant stakeholder groups as panel members and they contributed to the different steps of the process, including prioritization of recommendations and outcomes, evidence gathering, and adolopment of recommendations. Representation from the different Saudi regions and type of practice (governmental and private) enriched the discussions and contributed to enhancing the applicability of the recommendations. In addition, the inclusion of two international experts from the source guideline provided the rest of the panel with unique insight into the context of the source guideline and the source panel’s judgments.

The use of the GRADE-ADOLOPMENT methodology allowed the contextualization of guidelines in a relatively short period of time. A major facilitator was the use of the same methodology (i.e., GRADE) and tools (i.e., GRADEPro GDT) used in the source guideline. In addition, the use of the EtD framework facilitated structured discussions, and ensured that the panel considered both evidence on health effects and contextual data. While we collected primary cost data from the KSA context, we did not do so for the remaining contextual factors and had to rely on expert evidence provided by the panelists. This could have been achieved through either quantitative surveys [22, 23] or mixed methods studies [24].

In this project, none of the five adoloped recommendations were modified from the source recommendations in terms of strength. Two out of the five adoloped recommendations were modified in terms of direction. In a previous adolopment effort of the 2015 ACR treatment guideline for RA, five out eight adoloped recommendations were modified, specifically in terms of their strengths [2, 4].

The factors driving modification in this project were balance of effects (*n* = 2), valuation of outcomes (*n* = 1), cost (*n* = 1), and acceptability (*n* = 1). These factors, in addition to impact on health equity, were also driving factors for modification of recommendations in two other guideline adolopment efforts in the region [2, 5].

The evaluation scores on the PANELVIEW instrument were high when compared with scores for eight other panels [20]. When comparing the scores that our panel gave to the different items, they were the highest (6.85 to 6.62) for items under the domains of administration, methodology and process, group interaction and overall satisfaction. The lowest scores (6.08) were given to the item addressing the adequacy of time allocated to the panel meeting, and to the item addressing the opportunity to be involved in the prioritization of questions and scoping of the guideline.

With the increasing move towards living guidelines, groups adapting recommendations must deal with the challenge of frequently updated recommendations [25, 26]. The methodology addressing this situation has not yet been discussed or explored. Furthermore, while the list of adaptation-relevant prioritization criteria that we developed has face validity, more work is needed to refine and validate it. Finally, there is a need to develop methods for efficient collection of primary contextual data that would allow their consideration in adaptation projects with very short timelines.

## Conclusion

The GRADE-ADOLOPMENT methodology proved to be efficient. The panel positively assessed the process and outcome of this adolopment effort. The engagement of stakeholders with wide representation in terms of groups, regions and type of practice proved to be important for the success of this project.

### Supplementary Information

**Additional file 1. **Detailed timeline for the adolopment project.

**Additional file 2. **Survey data

**Additional file 3. **PANELVIEW instrument scores.

## Data Availability

All data generated or analysed during this study are included in this published article and its supplementary information files.

## Abbreviations

ACR: American College of Rheumatology
bDMARD: Biologic Disease-Modifying Anti-Rheumatic Drug
csDMARD: Conventional synthetic Disease-Modifying Anti-Rheumatic Drug
EtD: Evidence to Decision
EULAR: European League Against Rheumatism
G6PD: Glucose-6-Phosphate Dehydrogenase
GRADE: Grading of Recommendations Assessment, Development and Evaluation
HCQ: Hydroxychloroquine
KSA: Kingdom of Saudi Arabia
MTX: Methotrexate
RA: Rheumatoid Arthritis
SSR: Saudi Society for Rheumatology
SSZ: Sulfasalazine
tsDMARD: Targeted synthetic Disease-Modifying Anti-Rheumatic Drugs
