# Supplementary material for: Methodology for the adolopment of recommendations for the treatment of rheumatoid arthritis in the Kingdom of Saudi Arabia
Source: BMC Med Res Methodol. 2023 Oct 10;23:224. doi: 10.1186/s12874-023-02031-2 (PMC10563247; doi:10.1186/s12874-023-02031-2)
Supplement: Supplementary file 1 — Additional file 1. Detailed timeline for the adolopment project. [file 12874_2023_2031_MOESM1_ESM.docx]

**Additional file 1:** Detailed timeline for the adolopment project

|  | October 2021 | November 2021 | | | | December 2021 | | | | | January 2022 | | | | February 2022 | | | | March 2022 | | | | |  |  |  |
| --- | --- | --- | --- | --- | --- | --- | --- | --- | --- | --- | --- | --- | --- | --- | --- | --- | --- | --- | --- | --- | --- | --- | --- | --- | --- | --- |
|  |  | W1 | W2 | W3 | W4 | W1 | W2 | W3 | W4 | W5 | W1 | W2 | W3 | W4 | W1 | W2 | W3 | W4 | W1 | W2 | W3 | W4 | W5 |  |  |  |
| Panel selection |  |  |  |  |  |  |  |  |  |  |  |  |  |  |  |  |  |  |  |  |  |  |  |  |  |  |
| Question prioritization |  |  |  |  |  |  |  |  |  |  |  |  |  |  |  |  |  |  |  |  |  |  |  |  |  |  |
| Finalization of question selection |  |  |  |  |  |  |  |  |  |  |  |  |  |  |  |  |  |  |  |  |  |  |  |  |  |  |
| Declaration of conflicts of interest (COI) |  |  |  |  |  |  |  |  |  |  |  |  |  |  |  |  |  |  |  |  |  |  |  |  |  |  |
| Online panel meeting #1: Project introduction, planning, and panel training |  |  |  |  |  |  |  |  |  |  |  |  |  |  |  |  |  |  |  |  |  |  |  |  |  |  |
| Collection of cost data |  |  |  |  |  |  |  |  |  |  |  |  |  |  |  |  |  |  |  |  |  |  |  |  |  |  |
| Preparation of Evidence-to-Decision tables |  |  |  |  |  |  |  |  |  |  |  |  |  |  |  |  |  |  |  |  |  |  |  |  |  |  |
| Completion of PANELVoice for online panel meeting #2 |  |  |  |  |  |  |  |  |  |  |  |  |  |  |  |  |  |  |  |  |  |  |  |  |  |  |
| Online panel meeting #2: Formulation of adapted recommendations #1 and #2 |  |  |  |  |  |  |  |  |  |  |  |  |  |  |  |  |  |  |  |  |  |  |  |  |  |  |
| Completion of PANELVoice for online panel meetings #3 and #4 |  |  |  |  |  |  |  |  |  |  |  |  |  |  |  |  |  |  |  |  |  |  |  |  |  |  |
| Online panel meeting #3: Formulation of adapted recommendations #3 and #4 |  |  |  |  |  |  |  |  |  |  |  |  |  |  |  |  |  |  |  |  |  |  |  |  |  |  |
| Online panel meeting #4: Formulation of adapted recommendations #5 |  |  |  |  |  |  |  |  |  |  |  |  |  |  |  |  |  |  |  |  |  |  |  |  |  |  |
| Drafting guideline report |  |  |  |  |  |  |  |  |  |  |  |  |  |  |  |  |  |  |  |  |  |  |  |  |  |  |
| Review of guideline report by from panelists and finalization of report |  |  |  |  |  |  |  |  |  |  |  |  |  |  |  |  |  |  |  |  |  |  |  |  |  |  |
| Evaluation of adaptation process (administration of PANELView survey) |  |  |  |  |  |  |  |  |  |  |  |  |  |  |  |  |  |  |  |  |  |  |  |  |  |  |
