# Supplementary material for: Methodology for the adolopment of recommendations for the treatment of rheumatoid arthritis in the Kingdom of Saudi Arabia
Source: BMC Med Res Methodol. 2023 Oct 10;23:224. doi: 10.1186/s12874-023-02031-2 (PMC10563247; doi:10.1186/s12874-023-02031-2)
Supplement: Supplementary file 3 — Additional file 3. PANELVIEW instrument scores. [file 12874_2023_2031_MOESM3_ESM.docx]

**Additional file 3:** PANELVIEW instrument scores

| **Domain** | **Item** | **Mean score ± SD*** | **Range*** |
| --- | --- | --- | --- |
| 1. Administration | 1. The logistical support provided for organization of the guideline project and panel meeting was appropriate (e.g., scheduling of meeting) | 6.62 **±** 0.65 | 5-7 |
|  | 2. There was adequate preparatory work and meetings/teleconferences prior to the final panel meeting. | 6.38 **±** 0.65 | 5-7 |
|  | 3. Adequate time was given for guideline group members to complete tasks (e.g., surveys, providing feedback) throughout the development of the guideline, and to review the evidence summary and other material prior to the panel meeting. | 6.38 **±** 0.65 | 5-7 |
|  | 4. Adequate time was allotted for the final panel meeting for all guideline questions to be discussed and recommendations to be formulated. | 6.08 **±** 0.79 | 5-7 |
|  | 5. The panel meeting(s) had a clearly defined agenda and objectives. | 6.77 **±** 0.6 | 5-7 |
| 2. Training | 6. Information was provided about the specific methodology and frameworks to ensure understanding of the overall process and steps that would be used to develop the guideline. | 6.46 **±** 0.78 | 5-7 |
| 3. Panel chair | 7. The panel chair(s) was able to provide clinical and methodological guidance during the meeting, providing direction and support for decision-making. | 6.54 **±** 0.66 | 5-7 |
|  | 8. The panel chair(s) was able to manage the group process, establishing an atmosphere of support that ensured involvement of all panel members in the discussion and free expression of opinions. | 6.54 **±** 0.66 | 5-7 |
| 4. Conflict of interest | 9. There was appropriate management of potential interests (financial, academic) of guideline group members, of the organization, and in the evidence synthesis being free from bias. | 6.31 **±** 0.75 | 5-7 |
|  | 10. There was appropriate management of potential bias in panel members’ interpretation of evidence and alignment with prior beliefs. | 6.54 **±** 0.66 | 5-7 |
| 5. Scoping the guideline | 11. The panel was given sufficient opportunity to be involved in the prioritization of questions and scoping of the guideline. | 6.08 **±** 1.38 | 2-7 |
|  | 12. The final scope of the guideline was clearly communicated to the guideline development group and agreement was sought. | 6.54 **±** 0.52 | 6-7 |
| 6. Methodology and process | 13. The evidence synthesis was rigorous. | 6.46 **±** 0.66 | 5-7 |
|  | 14. A transparent and usable summary of the evidence was made available for the panel discussion. | 6.69 **±** 0.48 | 6-7 |
| 7. Considering the evidence and contributing through expertise | 15. Appropriate consideration was given to the evidence, including all relevant types, and balanced with panel members’ input and opportunity to use their experience to interpret the evidence. | 6.54 **±** 0.52 | 6-7 |
|  | 16. The method or process used for decision making with the available evidence was appropriate: | 6.38 **±** 0.65 | 5-7 |
|  | 17. There was appropriate involvement and consultation with key stakeholders during the guideline development. | 6.31 **±** 1.11 | 3-7 |
|  | 18. Appropriate consideration was given to patients’ views, perspectives, values and preferences. | 6.15 **±** 0.99 | 4-7 |
| 8. Formulating the recommendations | 19. An appropriate method was used for formulating the recommendations with transparency of judgements made. | 6.46 **±** 0.66 | 5-7 |
|  | 20. Appropriate consideration was given to relevant external factors (e.g., policy implications, setting-specific healthcare factors, acceptability of recommendations) in formulating the guideline recommendations. | 6.54 **±** 0.52 | 6-7 |
|  | 21. The consensus method used by the panel was appropriate, allowing ability to reach consensus. | 6.54 **±** 0.52 | 6-7 |
|  | 22. The wording of the guideline recommendations formulated was clear and actionable. | 6.54 **±** 0.66 | 5-7 |
|  | 23. There was transparency in going from the panel’s recommendation to the final recommendations that appear in the guideline report and notice was given about any changes made. | 6.54 **±** 0.52 | 6-7 |
| 9. Group composition | 24. There was diversity in membership and adequate representation of backgrounds, specialties and balance of expertise in the panel composition. | 6.54 **±** 0.52 | 6-7 |
|  | 25. The panel size was appropriate. | 6.54 **±** 0.52 | 6-7 |
| 10. Group roles | 26. The required commitment was at an appropriate level for the guideline group members. | 6.54 **±** 0.52 | 6-7 |
|  | 27. The contributions of the guideline group members were valued and appropriate credit was given. | 6.54 **±** 0.52 | 6-7 |
| 11. Group interaction | 28. There was mutual respect between guideline group members with friendly and professional conduct. | 6.69 **±** 0.48 | 6-7 |
| 12. Implementation and dissemination planning | 29. Appropriate consideration was given to the discussion of research gaps and needs for future research. | 6.31 **±** 0.75 | 5-7 |
|  | 30. Appropriate consideration was given for the planning of dissemination and implementation of the guideline. | 6.15 **±** 1.41 | 2-7 |
| 13. Writing guideline | 31. The writing of the guideline was well planned, with agreement on the format(s) and opportunity for panel members to provide input and review the guideline draft. | 6.38 **±** 0.51 | 6-7 |
| 14. Incentive | 32. I felt that my involvement in the guideline will have an impact on the health of people. | 6.46 **±** 0.88 | 4-7 |
| 15. Overall satisfaction | 33. Overall, I was satisfied with the guideline development process. | 6.46 **±** 0.52 | 6-7 |
|  | 34. I would participate in this guideline development process again. | 6.85 **±** 0.38 | 6-7 |

SD = standard deviation

*On a Likert scale ranging from 1 to 7
